# Knockdown of long noncoding RNA GAS5 reduces vascular smooth muscle cell apoptosis by inactivating EZH2-mediated RIG-I signaling pathway in abdominal aortic aneurysm

**DOI:** 10.1186/s12967-021-03023-w

**Published:** 2021-11-15

**Authors:** Tianming Le, Xin He, Jianhua Huang, Shuai Liu, Yang Bai, Kemin Wu

**Affiliations:** 1 Department of General and Vascular Surgery, Xiangya Hospital, Central South University, No. 87, Xiangya Road, Kaifu District, Changsha, 410008 Hunan Province People’s Republic of China; 2 Department of Anesthesiology, Xiangya Hospital, Central South University, Changsha, 410008 People’s Republic of China

**Keywords:** Abdominal aortic aneurysm, Smooth muscle cells, Apoptosis, GAS5, EZH2, RIG-I

## Abstract

**Background:**

Abdominal aortic aneurysm (AAA), an irreversible cardiovascular disease prevalent in the artery, causes the increase of the aneurysm diameter over time, and is a fatal phenomenon inducing sidewall rupture. Long noncoding RNAs (lncRNAs) serve as promising biomarkers for AAA. In the present study, we sought to define the role of lncRNA growth-arrest-specific transcript 5 (GAS5) in growth of smooth muscle cells (SMC) and progression of AAA.

**Methods:**

Initially, we established angiotensin II (Ang II)-induced AAA mouse models and Ang II-treated vascular SMC model. RT-qPCR and Western blot analysis were adopted to determine expression of GAS5 and zeste homolog 2 (EZH2). After ectopic expression and depletion experiments in Ang II-treated mice and vascular SMCs, cell apoptosis was detected in SMCs using flow cytometry and in mice using TUNEL staining. The binding of GAS5 and EZH2 was evaluated using RNA binding protein immunoprecipitation (RIP) and Co-IP assays.

**Results:**

Increased GAS5 and RIG-I but decreased EZH2 were found in aortic tissues of AAA mice. EZH2 overexpression inhibited AAA formation and suppressed SMC apoptosis. Functionally, EZH2 blocked the RIG-I signaling pathway and consequently inhibited SMC apoptosis. GAS5 regulated EZH2 transcription in a negative manner in SMCs. Knockdown of GAS5 attenuated SMC apoptosis, which was reversed by EZH2 inhibition or RIG-I overexpression.

**Conclusions:**

The current study demonstrated that GAS5 induced SMC apoptosis and subsequent AAA onset by activating EZH2-mediated RIG-I signaling pathway, highlighting GAS5 as a novel biomarker for AAA.

**Supplementary Information:**

The online version contains supplementary material available at 10.1186/s12967-021-03023-w.

## Background

Abdominal aortic aneurysm (AAA) is a prevalent irreversible cardiovascular disease occurring in the artery and the increase of the aneurysm diameter over time is a fatal phenomenon inducing sidewall rupture [1]. AAA represents a life-threatening vascular disease manifested by abdominal aorta dilation [2]. AAA is associated with chronic inflammation, vascular SMC apoptosis, extracellular matrix remodeling and degradation, and luminal thrombosis [3]. Patients succumbed to AAA often exhibit atheroembolization, abdominal or back pain, thromboembolization, aortic rupture, or development of an arteriovenous or aortoenteric fistula [4]. Numerous factors have been identified as the causative factors for AAA, including age older than 60 years, smoking, hypertension and Caucasian ethnicity [5]. The current treatment approaches for AAA are generally comprised of pharmacological and surgical approaches [6, 7]. Investigators focus more attention on non-coding RNAs (ncRNA) to AAA development as therapeutic targets or as biomarkers [8]. Understanding how ncRNAs contribute to AAA might thus suggest more specific and sensitive methods to describe disease process and to diagnose the disease at an early stage in a reliable manner [9]. Long ncRNAs (lncRNAs) are a kind of ncRNAs, and have attracted increasing research attention especially with regard to their regulatory action on proliferation, migration and apoptosis of vascular smooth muscle cells (SMCs) in AAA [10]. Dysfunction of vascular SMCs and other pathological cellular processes, such as inflammatory and immune responses, and vascular extracellular matrix remodeling, can contribute to AAA formation, and has been shown to be associated with LncRNAs, such growth-arrest-specific transcript 5 (GAS5), H19, Lnc-HLTF-5, HIF1α-as1 [8, 11].

LncRNA GAS5 is implicated in numerous diseases, including cardiovascular diseases [12, 13]. GAS5 has been shown to induce AAA formation by acting as a sponge for microRNA-21 to block Akt activation and phosphorylation and consequently to enhance apoptosis of SMCs [14]. In addition, knockdown of GAS5 has the potential to prevent the progression of atherosclerosis by reducing enhancer of zeste homolog 2 (EZH2)-mediated ATP-binding cassette transporters A1 (ABCA1) transcription [15]. EZH2 is an essential factor for homeostasis maintenance in postmitotic cardiomyocytes and for correct differentiation of cardiac progenitor cells [16]. EZH2 has been documented to inhibit retinoic acid-inducible gene-I (RIG-I) signaling pathway that is an essential signaling molecule to defend pathogen infections [17] in a methyltransferase-independent manner [18]. Previous data have flagged the potential of RIG-I to promote activation of downstream osteogenic mineralization responses in aortic SMCs by upregulating osteogenic genes [19]. Of note, vascular microcalcification has been identified to be indicative of AAA expansion [20]. Concurrently, RIG-I may play a deleterious role in AAA [21]. Therefore, we aimed at investigating whether GAS5 may affect AAA and the underlying mechanism involving the regulation of EZH2 and RIG-I. We hypothesized that GAS5 may promote SMC apoptosis and subsequent AAA onset by activating EZH2-mediated RIG-I signaling pathway.

## Materials and methods

### Ethics statement

The study was approved by the Ethics Committee of Xiangya Hospital and carried out in accordance with the Guide for the Care and Use of Laboratory animals published by the US National Institutes of Health. Extensive efforts were made to minimize the number and suffering of the included animals.

### Establishment of angiotensin II (Ang II)-induced mouse AAA models

C57BL/6 J male ApoE^−/−^ mice (aged 6–8 weeks and weighing 18–23 g; n = 12 in each group) were selected in this study. A small incision was made where osmotic micro-pump (Alzet, 2004; DURECT Corporation, Cupertino, CA, USA) was subcutaneously implanted into the back of mouse neck following anesthesia. Ang II (A9525, Sigma, St. Louis, MO, USA) or normal saline (0.9% NaCl) was injected through micropump (1 μg/kg per min). On the 28th day after Ang II infusion, the surviving mice were euthanized and the aortic tissues were extracted for further analysis, among which six of them were used for pathological examination and six for molecular biology detection [22].

### Lentivirus injection

Mice were injected intravenously via caudal vein with (2 × 10^12^ virus genome particles; n = 12 in each group) lentiviruses carrying negative control (NC), overexpression (oe)-EZH2, short hairpin RNA against GAS5 (sh-GAS5), sh-EZH2 and oe-RIG-I (Shanghai Genechem Co., Ltd., Shanghai, China) using an insulin syringe (BD Biosciences, Franklin Lakes, NJ, USA). After 30 days, the injected mice were used to develop AAA models [23]. After 28 days of modeling, the rats were euthanized for follow-up detection, including six for pathological detection and six for molecular biology detection.

### Aneurysm quantification

Six mice in each group were euthanized and abdominal incision was made to detect the presence of aortic aneurysm. To expose the aorta, we injected 10 mL of phosphate-buffered saline (PBS) from the left ventricle and overflowed from the incision in the right atrium. Under a dissecting microscope, the abdominal aorta was separated from the surrounding connective tissues and the connective tissues remaining on the adventitia of the abdominal aorta was removed with tweezers, with a full-length abdominal aorta photographed by a digital camera. The outer diameter of the abdominal aorta above the bifurcation of the renal artery was directly measured using images.

The maximum outer diameter of the abdominal aorta above the bifurcation of the renal artery was measured with Image-Pro Plus software [14]. The maximum outer diameter was measured at least three times and the average value was taken. The outer diameter of the aorta that increased by over 50% relative to that of the aorta injected with saline was regarded to be indicative of AAA.

### Immunohistochemistry

Aortic specimens were fixed, embedded, and cut into 5-µm-thick sections. The paraffin sections were then heated in a 60 °C oven for 30 min, routinely dewaxed, hydrated for 5 min each, and washed with water for 2 min. Next, the sections were subjected to antigen retrieval using 1 mM Tris-ethylene diamine tetraacetic acid (pH 8.0), washed with PBS for three times (5 min/time), immersed in 3% H_2_O_2_-formaldehyde at ambient temperature for 10 min, and washed with PBS for two times (5 min/time). Next, the sections were probed with primary antibody to EZH2 (1:100, ab191080, Abcam Inc., Cambridge, UK) overnight at 4 °C, washed with 0.1% Tween-20 for three times (5 min/time), and re-probed with poly-enhancer (PV-9000, ZSGB-Bio, Beijing, China) at ambient temperature for 20 min, followed by three washes using PBS containing 0.1% Tween-20 (5 min/time). Subsequently, the sections were further incubated with enzyme-labeled anti-mouse/rabbit polymer (PV-9000, ZSGB-Bio) at ambient temperature for 30 min. After washing with 0.1% Tween-20 for three times (5 min/time), the sections were developed using 3,3′-diaminobenzidine (DAB), counterstained and blued. At last, the sections were dehydrated, cleared, mounted, and observed under an inverted microscope (CX41, Olympus Optical Co., Ltd., Tokyo, Japan). Six non-overlapping fields were randomly selected (40 ×). Image J software was used to count the number of positively-stained cells and total cells. Every six tissue sections were selected from each group to calculate the percentage of cells positive for EZH2, followed by data analysis [24].

### Primer cell culture and grouping

Mouse primary vascular SMCs were cultured as previously described [25]. Briefly, mouse aorta (except endothelium and perivascular connective tissue and fat) was isolated and the vascular tissues were cut into 1-mm-thick pieces, treated with 1.42 mg/mL collagenase II for 4–6 h, and then cultured in Dulbecco’s modified Eagle’s medium containing 10% fetal bovine serum, streptomycin (100 μg/mL) and penicillin (100 U/mL) at 37 °C. Thereafter, the cells were treated with Ang II (10^–7^ mol/L) 24 h after lentivirus treatment at 37 °C. All cells were then incubated at 37 °C for subsequent experiments.

The cells were transfected with oe-NC, oe-EZH2, oe-EZH2 + oe-RIG-I, oe-GAS5 and oe-GAS5 + oe-EZH2 vectors (GenePharma Co., Ltd., Shanghai, China) and then treated with Ang II. Moreover, the cells transfected with only oe-NC were used as the controls.

### Terminal deoxynucleotidyl transferase (TdT)-mediated deoxyuridine triphosphate-biotin nick end labeling (TUNEL) assay

The abdominal aorta of mice was immersed in 0.85% sodium chloride solution, washed, and fixed with 3.4% paraformaldehyde or PBS solution containing 10% formalin for 15 min. Next, the samples were incubated in 100 μL protease K working solution (20 μg/mL) composed of 50 mmol/L TRIS–CL (pH 8.0) + 1.5 mmol/L calcium acetate + proteinase K (final concentration 20 mg/mL) for 10–30 min. After washing, the samples were fixed with 7.4% paraformaldehyde or PBS solution containing 10% formalin and then added with 100 μL balanced buffer solution. Next, the samples were put on ice to remove excess liquid and added with 100 μL TdT enzyme reaction solution for incubation for 60 min. Thereafter, the samples were immersed in PBS containing 0.3% H_2_O_2_ to neutralize the endogenous peroxidase. After PBS washing, the samples were added with 100 μL Streptavidin horseradish peroxidase for 30 min of reaction and treated with 100 μL DAB until the samples turned light brown. The samples were rinsed with deionized water, sealed, and observed under a microscope.

### Flow cytometry

Cell apoptosis and necrosis were evaluated using flow cytometry after staining with phycoerythrin-conjugated annexin V and 7-Aminoactinomycin D (BD Pharmingen, San Diego, CA, USA). The cells were then analyzed on a FACScan flow cytometer (Becton Dickinson, Mountain View, CA, USA) using the Cell Quest software (Becton Dickinson) and expressed as the percentage of positive cells.

### RNA binding protein immunoprecipitation (RIP) assay

The binding between GAS5 and EZH2 was evaluated using a Magna RIP TM RIP kit (Millipore, Billerica, MA, USA). Mouse SMCs were washed with pre-cooled PBS and scraped in radioimmunoprecipitation assay (RIPA) lysis buffer (P0013B, Beyotime, China) on ice for 5 min. The cells were then centrifuge at 14,000 rpm for 10 min at 4 °C, and part of the cell extract was absorbed as input, and the others were incubated with antibodies. For each co-precipitation reaction system, 50 µL magnetic beads were resuspended in 100 µL RIP Wash Buffer (EHJ-BVIS08102 Xiamen Jiahui Biotechnology Co., Ltd., China), and incubated with 5 µg antibody against EZH2 (#5246, 1:300, Cell Signaling Technology, Beverly, MA, USA) or immunoglobulin G (IgG) (1:100, ab109489, Abcam). After the magnetic bead-antibody complex was washed, the complex was resuspended in 900 µL RIP Wash Buffer, and interacted with 100 µL cell extract at 4 °C overnight. The magnetic bead-antibody complex and Input were digested with proteinase K, from which RNA was extracted for subsequent polymerase chain reaction (PCR) detection.

### Co-immunoprecipitation (Co-IP) assay

SMCs were lysed using cell lysis solution on ice for 30 min, after which the cell lysate was collected into a 1.5 mL Eppendorf tube and centrifuged at 12,000×*g* and 4 °C for 15 min, with the supernatant collected. Then 50 μL of protein A and protein G beads were extracted to the tube and suspended to form a mixture of 100 μL beads, 10 μL of which was added to the cell lysate with EZH2 antibody (ab191080, 1:1000, Abcam) and incubated at 4 °C overnight for coupling. After IP reaction, centrifugation was carried out at 3000 rpm and 4 °C for 3 min, with the agarose beads to the bottom of the tube. At last, 15 μL of 2 × sodium dodecyl sulfate (SDS) loading buffer was added to the precipitate, and boiled for 5 min. After cooling, the precipitate was analyzed by Western blot analysis to detect the expression RIG-I.

### RNA isolation and quantitation

The total RNA was extracted using TRIzol reagent (16096020, Thermo Fisher Scientific Inc., Waltham, MA, USA). Then 5 µg of the extracted RNA was reversely transcribed into complementary DNA (cDNA) using Reverse Transcription Kit (K1622, Fermentas Inc., Ontario, CA, USA). Primer sequences for GAS5, EZH2 and RIG-I are shown in Additional file 1: Table S1. With β-actin as internal reference, reverse transcription quantitative PCR (RT-qPCR) was then performed using TaqMan MicroRNA Assay and TaqMan^®^ Universal PCR Master Mix and TaqMan Gene Expression Assays protocol (Applied Biosystems, Foster City, CA, USA). Glyceraldehyde-3-phosphate dehydrogenase (GAPDH) was employed as the loading control and the expression was calculated using 2^−△△Ct^ method). The result was normalized to mRNA expression of β-actin.

### Western blot analysis

The total protein was extracted using RIPA lysis buffer (R0010, Beijing Solarbio Science & Technology Co., Ltd., Beijing, China). The total protein was quantified using a bicinchoninic acid protein concentration detection kit (GBCBIO Technologies Inc., Guangzhou, China). Next, 40 μg protein was separated by SDS–polyacrylamide gel electrophoresis and transferred onto polyvinylidene fluoride membranes (Millipore). The membrane was blocked with 5% bovine serum albumin at room temperature and probed overnight at 4 °C with primary rabbit antibodies to EZH2 (1:1000, ab191080), RIG-I (1:1000, ab45428), cleaved Caspase3 (1:1000, ab2302), B-cell lymphoma-2 (Bcl-2) (1:1000, ab182858), Bcl-2 associated protein X (Bax) (1:1000, ab32503), and β-actin (1:10,000, ab8226). The aforementioned antibodies were purchased from Abcam. The following day, the membrane was re-probed with secondary goat anti-rabbit IgG (1:2000, ab97051, Abcam) at room temperature. The immunocomplexes on the membrane were visualized using enhanced chemiluminescence, followed by photograph using Image Quant LAS 4000C software (GE, USA). With β-actin as internal reference, the blots were scanned, quantified for pixel density by the optical density method, and analyzed by Image lab software.

### Statistical analysis

All data were processed using SPSS 21.0 statistical software (IBM Corp., Armonk, NY, USA) and presented as mean ± standard deviation. Data between two groups were analyzed using the unpaired *t*-test and data among multiple groups were analyzed by one-way analysis of variance (ANOVA) with Tukey’s post hoc tests. *p* < 0.05 was considered to be statistically significant.

## Results

### EZH2 affects AAA development

**Fig. 1:**
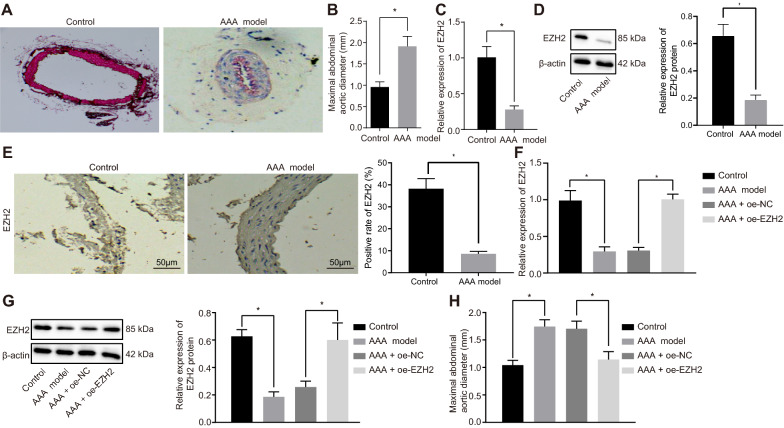
AAA mice present with low EZH2 expression and its upregulation attenuates AAA development.** A** The cross sections of abdominal aorta in AAA and control mice detected by HE staining. **B** The outer diameter of abdominal aorta of AAA and control mice. **C** The expression of EZH2 in aortic tissues of AAA and control mice determined by RT-qPCR. **D** Western blot analysis of EZH2 protein in aortic tissues of AAA and control mice. **E** Immunohistochemistry of EZH2 protein in aortic tissues of AAA and control mice (200 ×). **F** The expression of EZH2 in aortic tissues of control mice and AAA mice upon treatment with oe-NC and oe-EZH2 determined by RT-qPCR. **G** Western blot analysis of EZH2 protein in aortic tissues of control mice and AAA mice upon treatment with oe-NC and oe-EZH2. β-actin served as internal reference of EZH2. **H** Quantification of maximum abdominal aortic diameter of control mice and AAA mice upon treatment with oe-NC and oe-EZH2 *p < 0.05 indicates significant difference. Data in **A**–**H** were compared using unpaired *t*-test. n = 6 for mice following each treatment

We first set up an Ang II-induced AAA mouse model. The mice were euthanized 28 days after infusion of Ang II (AAA model) or equivalent saline (control), and the abdominal aorta was taken. AAA was visible to the naked eye in AAA model mice. Hematoxylin–eosin (HE) staining revealed flat, broken, and denatured middle layer of adventitia of some aneurysms, and thickened and re-shaped vessel wall in non aneurysmal part (Fig. 1A). The outer diameter of abdominal aorta increased by more than 50% in AAA mice (*p* < 0.05) (Fig. 1B). RT-qPCR and Western blot analysis depicted that EZH2 expression was decreased in aortic tissues of AAA mice when compared with the control mice (*p* < 0.05) (Fig. 1C, D). Immunohistochemistry results also showed a lower positive expression of EZH2 in aortic tissues of AAA mice than control mice (Fig. 1E). In order to check the role of EZH2 in AAA progression, we used lentivirus expressing oe-EZH2 whose mimicking activity was checked on aortic tissues of AAA mice (*p* < 0.05) (Fig. 1F, G). Using this technique, we found that AAA mice overexpressing EZH2 exhibited lower AAA formation rate than AAA mice, and the maximum abdominal aortic diameter was reduced in oe-EZH2-treated AAA mice (*p* < 0.05) (Fig. 1H). Thus, overexpression EZH2 could prevent the occurrence of AAA.

### Overexpression of EZH2 influences SMC apoptosis

**Fig. 2:**
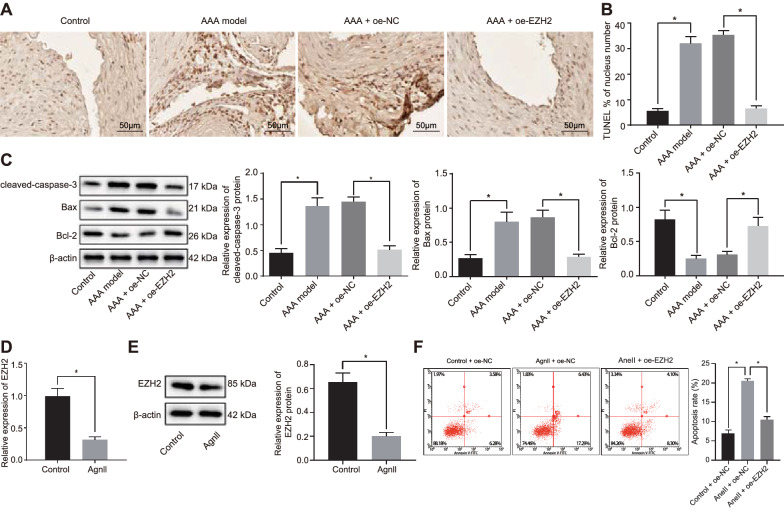
Upregulated EZH2 impedes SMC apoptosis. **A** Representative TUNEL staining image of SMC apoptosis in aortic tissues of control or AAA mice, and AAA mice treated with oe-EZH2 or oe-NC (400 ×). **B** Quantification of TUNEL-positive SMC ratio of cell apoptosis in aortic tissues control or AAA mice, and AAA mice treated with oe-EZH2 or oe-NC. **C** Western blot analysis of cleaved caspase-3, Bcl-2 and Bax proteins in abdominal aorta of AAA mice treated with oe-EZH2 or oe-NC. **D** The expression of EZH2 in Ang II-treated SMCs determined by RT-qPCR. **E** Western blot analysis of EZH2 protein in Ang II-treated SMCs. **F** Cell apoptosis in Ang II-treated SMCs overexpressing EZH2 measured by flow cytometry. β-actin served as internal reference of EZJ2 and cleaved-caspase-3. *p < 0.05 indicates significant difference. Data in **A**–**F** were compared using unpaired *t*-test. n = 6 for mice following each treatment. Cell experiments were repeated three times independently

SMC dysfunction and cell death contribute to dilatation and rupture of aorta, which is one of the important pathological features leading to AAA [26], so we herein detected SMC apoptosis in AAA mice upon treatment using TUNEL. It was evident that the SMC apoptosis rate of the abdominal aorta in the AAA mice relative to control mice was significantly increased, but the presence of oe-EZH2 in AAA mice significantly inhibited SMC apoptosis (*p* < 0.05) (Fig. 2A, B). Western blot analysis was performed to determine the protein levels of expression of apoptosis related genes cleaved caspase-3, Bax, and Bcl-2 in abdominal aorta of mice. The results illustrated an increase in protein expression of cleaved caspase-3 and Bax yet a decrease in Bcl-2 protein expression in aortic tissues of AAA mice compared with the controls, while a reduction in the protein expression of cleaved caspase-3 and Bax yet an increase in Bcl-2 protein expression in aortic tissues of AAA mice overexpressing EZH2 compared with AAA mice overexpressing NC (*p* < 0.05) (Fig. 2C). To further explore the effect of EZH2 on apoptosis of SMCs in vitro, we isolated SMCs from wild-type mice. Compared with the controls, SMCs treatment with Ang II showed decreased EZH2 expression (*p* < 0.05) (Fig. 2D, E). Flow cytometric data revealed that Ang II could induce cell apoptosis, which, however, was reduced by EZH2 overexpression (*p* < 0.05) (Fig. 2F). These results suggested that upregulated EZH2 could attenuate SMC apoptosis in AAA (Ang II induced in vitro).

### EZH2 regulates the RIG-I signaling pathway activation and thus affects SMC apoptosis

**Fig. 3:**
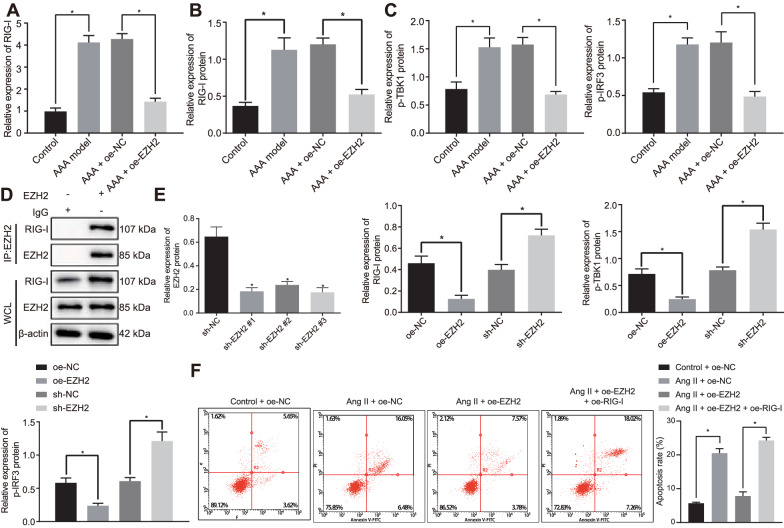
Upregulated EZH2 represses SMC apoptosis by blocking the RIG-I signaling pathway activation. **A** The expression of RIG-I in aortic tissues of control or AAA mice treated with oe-EZH2 and oe-NC detected by RT-qPCR. **B** Western blot analysis of RIG-I protein in aortic tissues of control or AAA mice treated with oe-EZH2 and oe-NC. **C** Western blot analysis of p-TBK1 and p-IRF3 proteins in aortic tissues of control or AAA mice treated with oe-EZH2 and oe-NC. **D** The interaction between RIG-I and EZH2 in SMCs assessed using Co-IP assay. *WCL* whole cell lysis. **E** Western blot analysis of RIG-I, p-TBK1 and p-IRF3 proteins in SMCs with oe-EZH2 or sh-EZH2. **F** Cell apoptosis in Ang II-induced SMCs co-treated with Ang II, oe-EZH2 and oe-RIG-I, or oe-NC measured by flow cytometry. β-actin served as internal reference of RIG-I and p-TBK1 *p < 0.05 indicates significant difference. Data in **A**–**C** and **E** were compared using unpaired *t*-test and in **F** by one-way ANOVA with Tukey’s post-hoc tests. n = 6 for mice following each treatment. Cell experiments were repeated three times independently

Next, we shifted our attention to determine how EZH2 functions in SMC apoptosis. Using RT-qPCR and Western blot analysis, we found an increase in RIG-I expression in AAA mice compared with the controls while it was downregulated in oe-EZH2-treated AAA mice comparted with oe-NC-treated AAA mice (*p* < 0.05) (Fig. 3A, B), suggesting EZH2 can inhibit RIG-I expression in AAA mice. Western blot analysis was conducted to further detect the expression of RIG-I signaling pathway downstream proteins TANK binding kinase 1 (TBK1) and interferon regulatory factor 3 (IRF3). We found that the extent of p-TBK1 and p-IRF3 was enhanced in abdominal aorta of AAA mice compared with the controls whereas it was reduced in abdominal aorta of AAA mice treated with oe-EZH2 comparted with oe-NC-treated AAA mice (*p* < 0.05) (Fig. 3C). Co-IP assay results demonstrated an interaction between EZH2 and RIG-I in SMCs (*p* < 0.05) (Fig. 3D). SMCs upon EZH2 overexpression had reduced RIG-I, extent of TBK1, and IRF3 phosphorylation, while those upon EZH2 silencing showed opposite trends as compared with NC (*p* < 0.05) (Fig. 3E), suggesting the inhibitory role of EZH2 in the RIG-I signaling pathway. Furthermore, as shown in Fig. 3F (*p* < 0.05), after Ang II treatment, cell apoptosis was attenuated following EZH2 overexpression compared with NC treatment, but it was reversed by further RIG-I overexpression. The aforementioned results suggested that EZH2 inhibited the apoptosis of SMCs by inactivating the RIG-I signaling pathway.

### The effects of GAS5 on the apoptosis of SMCs depends partially on the inhibition of EZH2

**Fig. 4:**
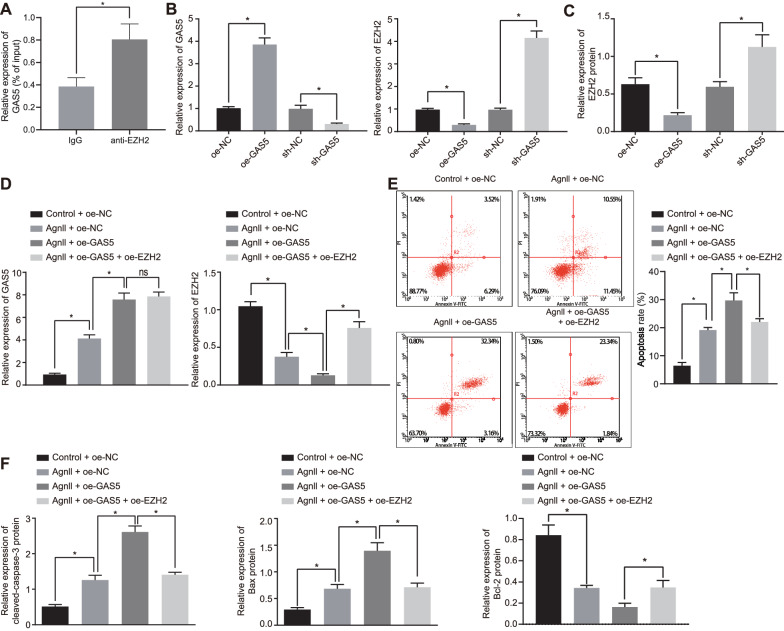
GAS5 promotes SMC apoptosis partially by downregulating EZH2 expression. **A** The interaction between GAS5 and EZH2 analyzed by RIP assay. **B** The expression of GAS5 and EZH2 in SMCs transfected with oe-GAS5 or sh-GAS5 detected by RT-qPCR. **C** Western blot analysis of EZH2 protein in SMCs transfected with oe-GAS5 or sh-GAS5. **D** Transfection efficiency of GAS5 and EZH2 in SMCs determined by RT-qPCR. **E** Cell apoptosis in Ang II-induced SMCs co-treated with oe-EZH2, oe-NC and oe-GAS5 or control mice treated with oe-NC measured by flow cytometry. **F** Western blot analysis of cleaved caspase-3, Bcl-2 and Bax proteins in Ang II-induced SMCs co-treated with oe-EZH2, oe-NC and oe-GAS5 or control mice treated with oe-NC. β-actin served as internal reference of EZH2 and cleaved-caspase-3. *p < 0.05 indicates significant difference. Data in **B** and **C** were compared using unpaired *t*-test while in **D**–**F** by one-way ANOVA with Tukey’s post-hoc tests. Cell experiments were repeated three times independently

It has been reported that GAS5 promotes the apoptosis of SMCs [14]. The results of RIP experiments firstly showed that compared with IgG, anti-EZH2 treatment elevated GAS5 expression, suggesting that GAS5 could bind to EZH2 (Fig. 4A). Furthermore, RT-qPCR and Western blot analysis demonstrated that EZH2 expression was diminished in oe-GAS5-treated SMCs compared with oe-NC, while it was upregulated in the absence of GAS5 compared with sh-NC (*p* < 0.05) (Fig. 4B, C). These results suggested that GAS5 negatively regulated EZH2 transcription in SMCs.

We hypothesized that the impact of GAS5 on the apoptosis of SMCs was partially dependent on its inhibition in EZH2 expression. To verify this, we altered GAS5 and EZH2 expressions in SMCs. RT-qPCR results showed that compared with control + oe-NC, treatment with Ang II + oe-NC elevated GAS5 expression. Compared with Ang II + oe-NC, treatment with Ang II + oe-GAS5 had elevated GAS5 expression and reduced EZH2 expression. Compared with Ang II + oe-GAS5, treatment with Ang II + oe-GAS5 + oe-EZH2 increased EZH2 expression but had no effect on GAS5 expression (*p* < 0.05) (Fig. 4D). Flow cytometric analysis showed that compared with Ang II + oe-NC, apoptosis of SMCs was enhanced by Ang II + oe-GAS5, which was partially abolished by further overexpression of EZH2 (*p* < 0.05) (Fig. 4E). In addition, increased expression of cleaved caspase-3 and Bax, yet decreased Bcl-2 expression were observed in SMCs after Ang II + oe-GAS5 treatment compared with Ang II + oe-NC, which were also partially eliminated by further overexpressed EZH2 (*p* < 0.05) (Fig. 4F). The above results suggested that GAS5 could augment SMC apoptosis by suppressing EZH2 expression.

### GAS5 regulates AAA development via the EZH2/RIG-I axis

**Fig. 5:**
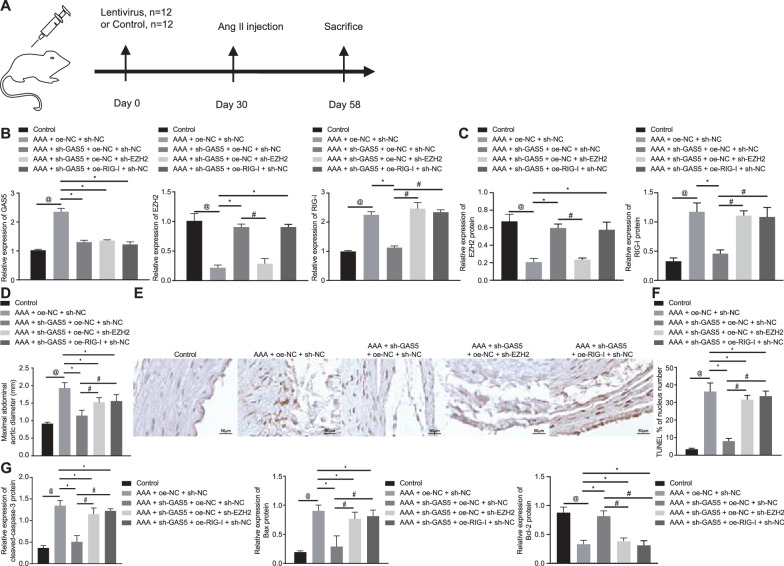
GAS5 promotes AAA progression through regulation of the EZH2/RIG-I axis. **A** Experiment flow chart. **B** The expression of GAS5, EZH2 and RIG-I in aortic tissues of control or AAA mice treated with sh-EZH2, sh-GAS5, oe-RIG-I, sh-NC and oe-NC detected by RT-qPCR. **C** Western blot analysis of EZH2 and RIG-I proteins in mouse aortic tissues. **D** The maximum abdominal aorta diameter of mice. **E** Representative TUNEL staining image of Cell apoptosis in mouse aortic tissues of control or AAA mice treated with sh-EZH2, sh-GAS5, oe-RIG-I, sh-NC and oe-NC (400 ×). **F** Quantification of TUNEL positive ratio of cell apoptosis in mouse aortic tissues. **G** Western blot analysis of cleaved caspase-3, Bcl-2 and Bax proteins in mouse aortic tissues. ^@^*p* < 0.05 vs. the control group. *p < 0.05 vs. the AAA + oe-NC + sh-NC group; ^#^*p* < 0.05 vs. the AAA + sh-GAS5 + oe-NC + sh-NC group. Data in **A**–**G** were compared using one-way ANOVA with Tukey’s post-hoc tests. n = 6 for mice following each treatment

To understand the interaction among GAS5, EZH2, and RIG-I in AAA in vivo, we established knockdown of GAS5 and EZH2 along with lentivirus expressing oe-RIG-I, and oe-NC. Then, the lentivirus was injected into AAA mice (Fig. 5A). RT-qPCR and Western blot analyses detected increases in the expression of GAS5 and RIG-I yet a downward trend regarding EZH2 expression in abdominal aorta of AAA mice treated with NCs compared with the controls. However, sh-GAS5-treated AAA mice exhibited increased EZH2 expression and decreased GAS5 and RIG-I expression compared with those treated with NCs. Additionally, AAA mice co-treated with sh-GAS5 and oe-RIG-I presented with augmented RIG-I expression and those co-treated with sh-GAS5 and sh-EZH2 had diminished EZH2 expression (*p* < 0.05) (Fig. 5B, C). It was evident that the maximum abdominal aorta diameter of sh-GAS5-treated AAA mice was reduced as compared with the NCs but the diameter was restored upon further treatment with sh-EZH2 or oe-RIG-1 (*p* < 0.05) (Fig. 5D). These results suggested that knockdown of GAS5 might restrict the development of AAA, which could be partially reversed by further knockdown of EZH2 or overexpression of RIG-I. TUNEL staining suggested a decline in cell apoptosis in response to GAS5 knockdown as compared with the NCs, while an increase was observed in response to further sh-EZH2 or oe-RIG-1 treatments (*p* < 0.05) (Fig. 5E, F). Furthermore, Western blot analysis showed that GAS5 silencing led to decreased cleaved caspase-3 and Bax expression and increased Bcl-2 expression as compared with the NCs, which was reversed in further presence of sh-EZH2 or oe-RIG-1 (*p* < 0.05) (Fig. 5G). These data showed that GAS5 deficiency could reduce cell apoptosis and alleviate progression of AAA, which was partially rescued by further knockdown of EZH2 or overexpression of RIG-I in AAA mice.

The above diagrams served to illustrate that GAS5 suppressed EZH2 expression and subsequently promoted the RIG-I signaling pathway, thus potentiating cell apoptosis and accelerating AAA progression.

## Discussion

Our study set out to delineate the effect of GAS5/EZH2/RIG-I axis on SMC apoptosis in AAA. The gathered findings revealed that GAS5 could activate the RIG-I signaling pathway by binding to EZH2 and consequently result in the apoptosis of SMCs, which promoted AAA progression.

Our initial results showed that EZH2 had a low expression in aortic tissues of AAA mice. Similar to our results, EZH2 is detected to be significantly downregulated in AAA tissues as recently reported [27]. EZH2 has been indicated in the pathogenesis of abnormal vasculature and vascular disease. For instance, mice with EZH2 knockout present increased cardiac growth hallmarked by elevated expression of hypertrophic and fibrotic genes [28]. In addition, EZH2 activation has preventive potential for aortic dissection as its overexpression inhibits autophagic cell death of aortic vascular SMCs [3]. Furthermore, inhibition of EZH2/1 is capable of significantly suppressing vascular SMC proliferation induced by platelet-derived growth factor-BB [29]. In consistent with these data, the present study revealed that overexpression EZH2 could prevent the occurrence of AAA and attenuate SMC apoptosis.

EZH2 has been shown to inhibit the activation of the RIG-I signaling pathway as evidenced by reduced extent of TBK1 and IRF3 phosphorylation, thereby repressing the replication of influenza A virus in cells [18]. Hence, this study sought out to further detect the activity of RIG-I signaling pathway. Previous data shows that RIG-I is preferentially expressed in both aortic tissues and blood samples from patients with AAA when compared to those from healthy volunteers, implicating the importance of RIG-I in AAA development [21]. RIG-I represents a stimulus for mitochondrial antiviral signaling (MAVS) capable of inducing cardiovascular calcification in human with Singleton-Merten syndrome and aortic calcium accumulation is found to be decreased in MAVS-deficient mice with arteriosclerosis [19]. Notably, TBK1, as a non-canonical inhibitor of nuclear factor kappa-B (NF-κB) kinase, is of importance to the NF-κB signaling pathway while phosphorylated TBK1 can phosphorylate IRF3 to trigger its dimerization and nuclear translocation, by which production of IRF1 and inflammatory cytokines is promoted in association with pathogenesis of multiple inflammatory diseases [30, 31]. Available data have elucidated that phosphorylated TBK1 and IRF3 can induce inflammatory response and apoptosis of vascular SMCs [32, 33]. Moreover, RIG-I drives the pathological process of intramural differentiation and migration of vascular SMCs which are involved in the development of arteriosclerosis [34]. Collectively, the aforementioned data indicate that EZH2 may restrain the apoptosis of SMCs by inactivating the RIG-I signaling pathway.

In the present work, we also found that EZH2 suppressed the activity of RIG-I signaling pathway, thus enhancing SMC apoptosis and promoting AAA progression. Besides, our data showed that GAS5 suppressed EZH2 expression and subsequently aggravated AAA. The adverse relationship between GAS5 and EZH2 has been extensively reported. More specifically, GAS5 has the potential to inhibit EZH2 transcription by directly interacting with E2F transcription factor 4 (E2F4) and recruiting E2F4 to the promoter of EZH2 in bladder cancer cells [35]. GAS5 binds to EZH2 and then inhibits the expression of ABCA1, thus accelerating the progression of atherosclerosis [15]. GAS5 suppresses Th1 differentiation and promotes Th2 differentiation via EZH2 inhibition, thus eliciting the development of allergic rhinitis [36]. Additionally, GAS5 has been demonstrated its suppressive effects on vascular SMC proliferation and promoting effects on cell apoptosis via activation the p53 signaling pathway in vascular remodeling [37]. A recent study has illustrated the functionality of GAS5 to contribute to AAA formation by promoting SMC apoptosis [14], which is in line with our findings. To data, several lncRNAs have been implicated in the regulation of RIG-I signaling pathway. Knockdown of lnczc3h7a has been suggested to hamper RIG-I signaling pathway and the subsequent antiviral innate responses to RNA viruses in vitro and in vivo [38]. LncRNA AFAP1-AS1 activates the RIG-I-like receptor signaling pathway in vitro, which enhances cancer cell migration and invasion in non-small cell lung cancer [39]. However, there is limited information on the role of GAS5 in the RIG-I signaling pathway, which merits further investigation.

## Conclusions

**Fig. 6:**
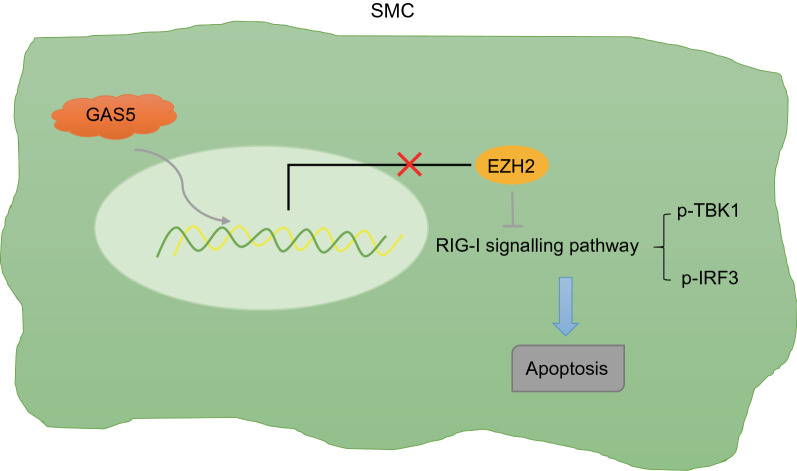
The mechanism graph of the regulatory network and function of GAS5 in AAA. GAS5 can mediate EZH2 transcription in a negative manner to activate the RIG-I signaling pathway, and then boost the apoptosis of SMCs, thus leading to the occurrence of AAA

The findings from the present study suggested GAS5 can play a promoting role in AAA through EZH2-dependent RIG-I enhancement (Fig. 6). Thus, the GAS5/EZH2/RIG-I axis may be potential future therapeutic strategies for AAA. However, future studies employing specimens from patients with AAA are required for the further analysis on the implication of the GAS5/EZH2/RIG-I axis in the clinical setting. Also, whether GAS5 contributes to AAA through other associated signaling pathways has not been illuminated, and the specific mechanism involving the GAS5/EZH2/RIG-I axis in cellular network alterations still needs to be identified through further studies.

## Supplementary Information

**Additional file 1: Table S1.** Primer sequences for RT-qPCR.

## Data Availability

The datasets generated and/or analysed during the current study are available from the corresponding author on reasonable request.

## Abbreviations

AAA: Abdominal aortic aneurysm
lncRNAs: Long noncoding RNAs
SMC: Smooth muscle cell
Ang II: Angiotensin II
EZH2: Zeste homolog 2
ncRNA: Non-coding RNAs
GAS5: Growth-arrest-specific transcript 5
miR: MicroRNA
ABCA1: ATP-binding cassette transporters A1
