# Supplementary material for: Knockdown of long noncoding RNA GAS5 reduces vascular smooth muscle cell apoptosis by inactivating EZH2-mediated RIG-I signaling pathway in abdominal aortic aneurysm
Source: J Transl Med. 2021 Nov 15;19:466. doi: 10.1186/s12967-021-03023-w (PMC8594130; doi:10.1186/s12967-021-03023-w)
Supplement: Supplementary file 1 — Additional file 1: Table S1. Primer sequences for RT-qPCR. [file 12967_2021_3023_MOESM1_ESM.docx]

**Additional file 1: Table S1.** Primer sequences for RT-qPCR

| Targets | Primer sequences (5’-3’) |
| --- | --- |
| GAS5 | F: 5’-AAGCCATTGGCACACAGGCATTAG-3’ |
|  | R: 5’-AGAACCATTAAGCTGGTCCAGGCA-3’ |
| EZH2 | F: 5’-AGCACAAGTCATCCCGTTAAAG-3’ |
|  | R: 5’-AATTCTGTTGTAAGGGCGACC-3’ |
| RIG-I | F: 5’-AAGCCATCGAAAGTTGGGACT-3’ |
|  | R: 5’-GCACCTGCCATTCTCCCTTTA-3’ |
| GAPDH | F: 5’-AGGTCGGTGTGAACGGATTTG-3’  R: 5’-GGGGTCGTTGATGGCAACA-3’ |

Note: RT-qPCR, reverse transcription quantitative polymerase chain reaction; F, forward; R, reverse; GAS5, growth arrest-special transcript 5; EZH2, enhancer of zeste homolog 2; RIG-I, retinoic acid-inducible gene-I; GAPDH, glyceraldehyde-3-phosphate dehydrogenase.
